# Stability of circulating microRNAs in serum

**DOI:** 10.1371/journal.pone.0268958

**Published:** 2022-08-31

**Authors:** Tomas Kupec, Andreas Bleilevens, Séverine Iborra, Laila Najjari, Julia Wittenborn, Jochen Maurer, Elmar Stickeler

**Affiliations:** Clinic for Gynaecology and Obstetrics, University Hospital RWTH Aachen, Aachen, Germany; Universitat des Saarlandes, GERMANY

## Abstract

There is a strong body of evidence by several translational studies which demonstrate the potential of circulating miRNAs as a potential biomarker in oncology. However, recent reports documented varying stability of these small RNA molecules in serum samples. The aim of our pilot study was to evaluate the stability of miRNAs in serum in relation to food intake and sample storage. Serum miRNA expression levels of 16 different miRNAs from 8 healthy volunteers were quantified by real-time PCR. 4 samples from each donor were analysed—2 samples (fasting, in the morning and after food intake, at noon) were analysed within 24h and 2 samples (fasting and after food intake, at noon) were stored at -80°C for 14 days and subsequently analysed. Student´s t-test was used to determine significant differences. The detectability of the distinct miRNA as a surrogate for the stability of these small RNA molecules was slightly altered by the storage conditions, but only a miRNA 22-3p, out of the analysed 16 miRNAs, shows significant lower dCq expression (3.821 vs. 4.530; p<0,01) by qPCR dependent on storage conditions (-80°C vs. 4°C). However, miRNA levels were not affected by food intake. The difference between samples taken in the morning (fasting) and at noon (after a normal meal) did not show any significant differences. MiRNAs can be considered to be a relatively stable tool in laboratory diagnostics, but clearly every new assay needs thorough evaluation. The stability of miRNAs documented here in healthy volunteers shows their potential in the search for innovative biomarkers in oncology.

## Introduction

In recent years, liquid biopsies have developed from a method of translational research to important tools in clinical practice. In oncology, in addition to the direct detection of tumour cells, the method focuses primarily on the blood-based analysis of nucleic acids, which enables the detection of tumour cells or cell free DNA. Potential areas of application are screening for early detection, therapy monitoring under systemic therapy, use as a prognostic biomarker and the detection of newly acquired mutations for optimized and targeted therapy selection. The great advantages of minimally invasive liquid biopsy over tissue biopsy are obvious, the latter is not always feasible and is also painful, time-consuming and invasive. Furthermore, liquid biopsy allows for repeated sample collection as monitoring of a disease [1].

MicroRNA (miRNA) represents a subtype of RNA, a family of small, 17–24 short, non-coding RNAs that post-transcriptionally regulate gene expression by gene silencing. They play a fundamental role in developmental stage-dependence as well as in cell differentiation, proliferation, apoptosis and tumorigenesis [2]. Circulating miRNAs are very stable and resistant to endogenous RNAses. Therefore, these molecules are potentially very well suited to be used as biomarkers for the detection or progression of tumour disease.

Sampling usually takes place at different times of the day and the serum samples are usually used for scientific purposes after being thawed from -80°C. For clinical use, it is important to determine the variation in microRNA stability and expression in serum with different storage conditions as well as different timepoints of collection.

The aim of this pilot study was to investigate the stability of circulating microRNAs in different conditions: First, short-term storage at 4°C and subsequent microRNA isolation and to compare their stability after storage of samples for 14 days at -80°C, subsequent thawing and microRNA isolation. Second, we compared the stability of microRNAs collected from the same volunteer in fasting condition in the morning and after several hours with an usual food and water intake.

These investigations replicate the issues we are exposed to in clinical practice during sample collection and subsequent processing. Without knowledge of the different stability and expression values of specific microRNAs under these conditions, their implementation into practice is not possible.

The aim of this pilot study was to examine changes in serum miRNAs according to different collection and storage conditions. We chose for these study 16 miRNAs which have been evaluated in breast cancer studies: 361-5p, 28-5p, 194-5p,125b-5p, 192-5p, 27b-3p, 151a-5p, 1260a, 100-5p, 151a-3p, 323b-5p, 99a-5p, 28-3p, 22-3p, 193a-5p, 193b-3p [3–17].

## Material and methods

### Sampling

Samples were taken from 8 volunteers by professional health care personal in the RWTH Uniklinik Aachen. In total 4 Serum tubes with 10 ml whole blood volume where collected. Two samples were collected in the morning with fasting condition (samples 1) and two samples in the afternoon (samples 2). There were no further regulations towards the afternoon samples due to food intake and drinking. Every sample was stored until fully coagulated. Afterwards samples were centrifuged by 2500 x g for 10 minutes. Serum supernatant was pipetted carefully and separated into aliquots. Aliquots were divided into two groups. One group of samples were lysed and miRNA was isolated on the same day (samples A) and the samples of the other group were stored for 14 days at -80°C (samples B). Samples of the group B have been isolated on day 15 after being stored at -80°C.

### RNA isolation

RNA isolation was performed with the miRNeasy Mini Kit by Qiagen (#217004) following the instructions of the user manual. Aliquots contained each 200μl of serum for isolation. After isolation the isolated RNA was stored at -80°C until cDNA transcription.

### Analysis of samples with quantitative PCR

#### Transcription

miRNA Samples were transcribed and amplified using multistep TaqMan Advanced cDNA Synthesis Kit (ThermoFisher, A28007). Steps were proceeded following the manufacturer’s instructions. As an exogenous control, the synthetic miRNA ath-miR-159a (ThermoFisher, 478411_mir/A25576), was inserted into the first step (poly-A tailing) of the synthesis kit in specific amount (6 pM). Samples could be stored after the last step at -20°C until further use. In advance of qPCR experiments, samples were diluted 1:10 with 0.1X TE buffer (1xTE pH 8,0 from PanReac AppliChem (A2575)) as recommended by the manufacturer’s instructions.

#### Quantitative PCR

Transcribed miRNA-Samples were analyzed using TaqMan Advanced miRNA Assay (#A25576 Applied Biosystems) in combination with TaqMan Fast Advanced Master Mix (#4444557 Applied Biosystems). The Roche LightCycler 480 Instrument II (#05015243001) was used for detection. Samples and master mix were applied to 384 well plates (#04729749001 LightCycler 480 Multiwell Plate white, Roche). For correct preparation following the manufacturer´s instructions, samples were diluted with 0.1x TE. Samples were pipetted in triplets (technical replicates) on each plate per miRNA-Assay as well as the exogenous control ath-mir-159a. We detected expression of hsa-miR-361-5p, hsa-miR-28-5p, hsa-miR-194-5p, hsa-miR-125b-5p, hsa-miR-192-5p, hsa-miR-27b-3p, hsa-miR-151a-5p, hsa-miR-1260a, hsa-miR-100-5p, hsa-miR-151a-3p, hsa-miR-323b-5p, hsa-miR-99a-5p, hsa-miR-28-3p, hsa-miR-22-3p, hsa-miR-193a-5p, hsa-miR-193b-3p using this setup.

### Statistics for qPCR

Data of LightCycler 480 were exported as MS-EXCEL files and analyzed. Resulting Ct (Cycle-times) Values were analyzed using ΔΔCt method. MicroRNA ath-mir-159a was used as reference gene to normalize data as ΔCt and samples from different times and storage conditions served as second reference to calculate miRNA fold change.

GraphPad Prism 9 software was used for statistical evaluation. Student’s t-test was used to determine significant differences.

The study was covered and enabled by the work of the Centralized Biomaterial Bank at RWTH Aachen. The protocol was approved by the independent ethics committee of the faculty of medicine at RWTH (see ethics vote 206/09). Written informed consent was obtained from each volunteer.

## Results

**Fig 1:**
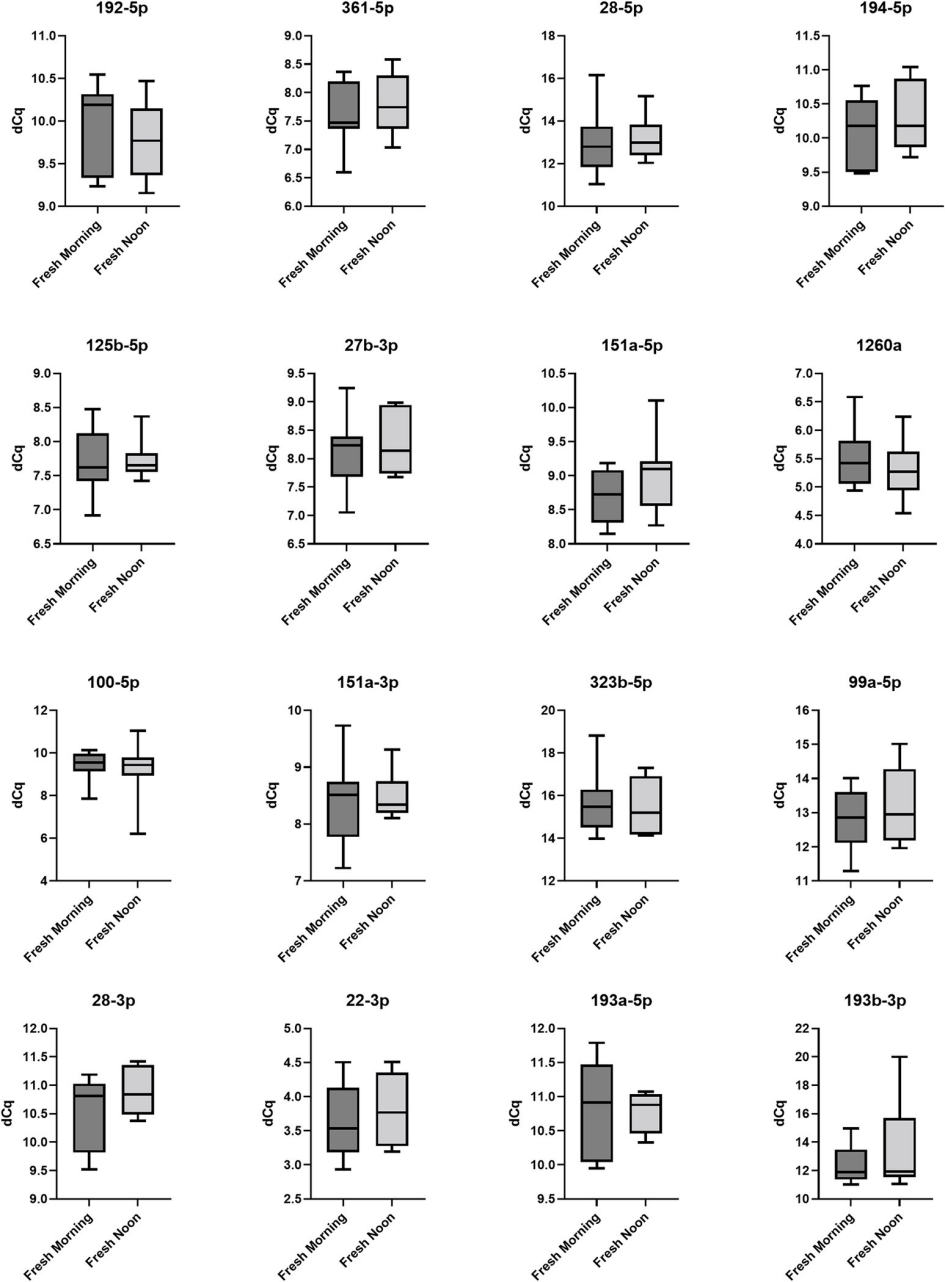
Fresh morning versus fresh noon. Box plots of dCq values of serum MiRNA samples (n = 8) taken in the morning under fasting condition compared to samples (n = 8) taken at noon after normal eating and drinking. All samples were analyzed within 24 hours. The differences in the expression were not significant.

**Fig 2:**
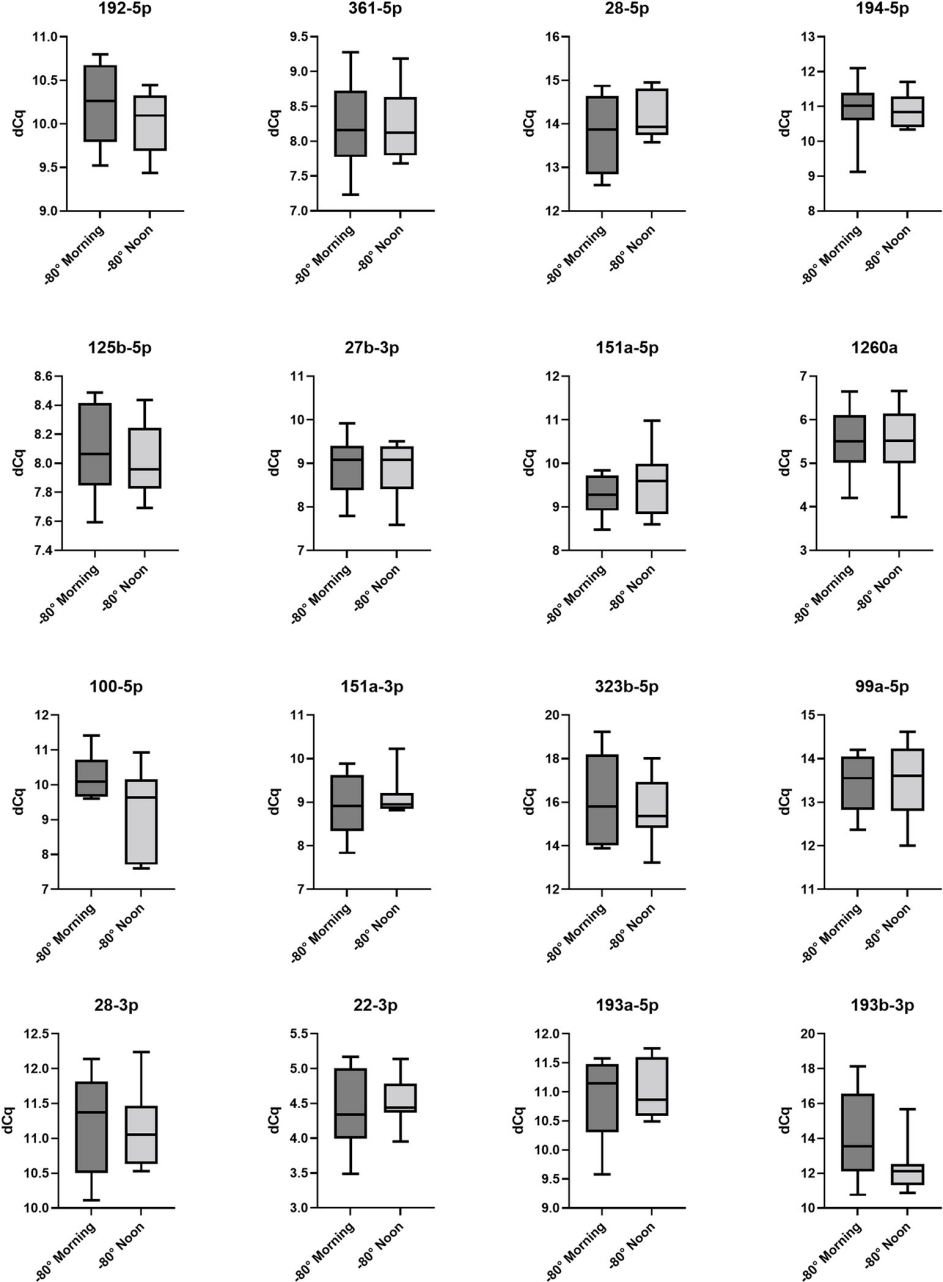
-80°C morning versus -80°C noon. Box plots of dCq values of serum MiRNA samples (n = 8) taken in the morning under fasting condition compared to samples (n = 8) taken at noon after normal eating and drinking. All samples were stored 14 days by -80°C and then analyzed. The differences in the expression were not significant.

In clinical practice, samples are often taken at different times of the day. Therefore, we wanted to analyse influence of time in the day and food intake on miRNA abundance in patient serum samples. Fig 1 shows how the miRNA assays, which were analysed on the same day varied after patients ate and drank normally for at least 6 hours. The complete panel of the selected 16 miRNAs was detectable in serum without any significant differences. The same findings were observed in the miRNA samples, which compare the analysed samples at different times of the day after 14 days storage at -80°C (Fig 2). We did not find any significant differences in expression.

**Fig 3:**
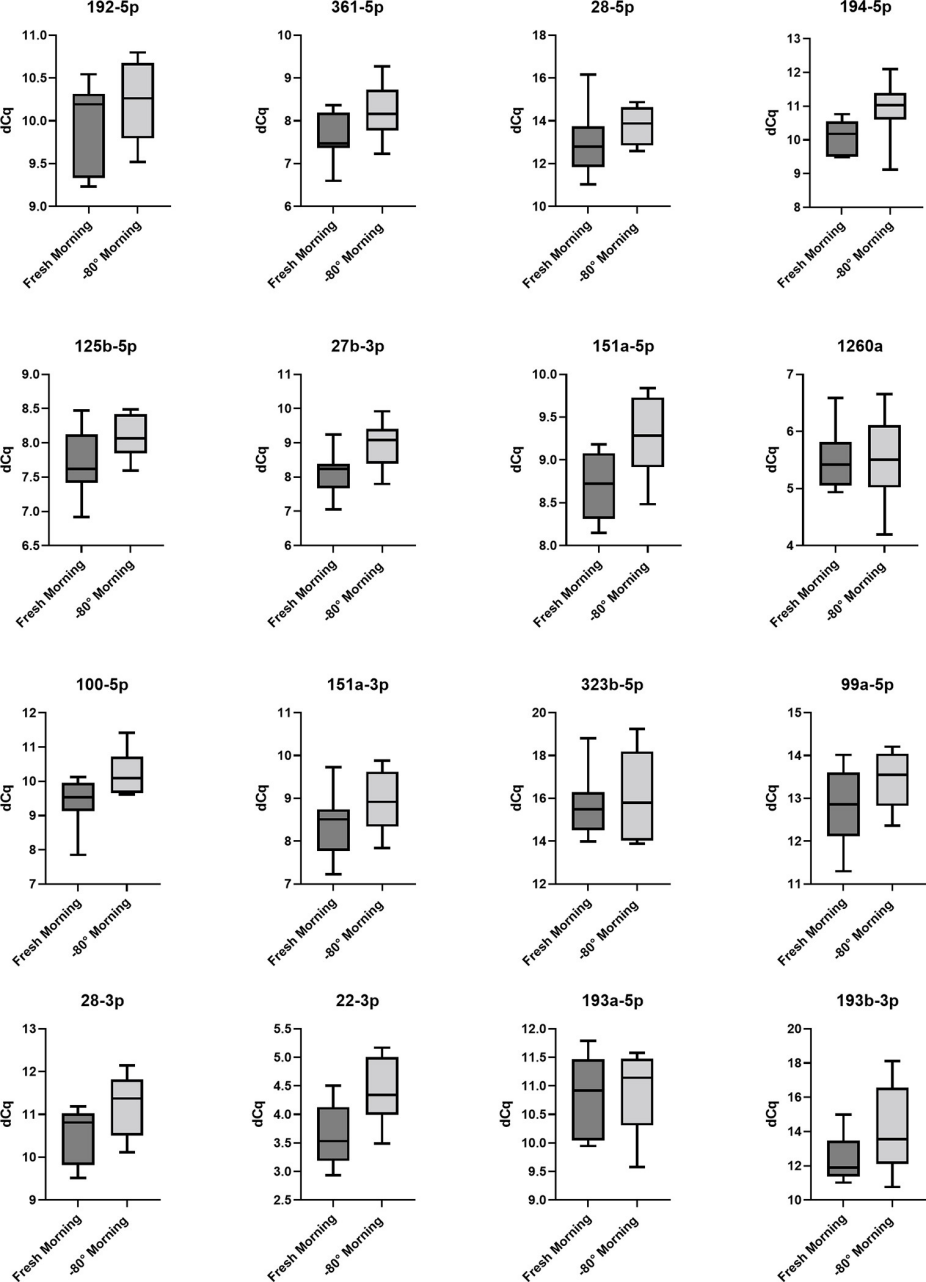
Fresh morning versus -80°C morning. Box plots of dCq values of serum MiRNA samples taken at fasting condition compares the analysis within 24 hours (n = 8) and the analysis of samples (n = 8) after 14 days storage at -80°C. The expression of miRNAs 194-5p, 27b-3p, 151a-5p, 100-5p, 22-3p was lower after being stored at -80° for 14 days. There were no significant differences (p<0,01).

**Fig 4:**
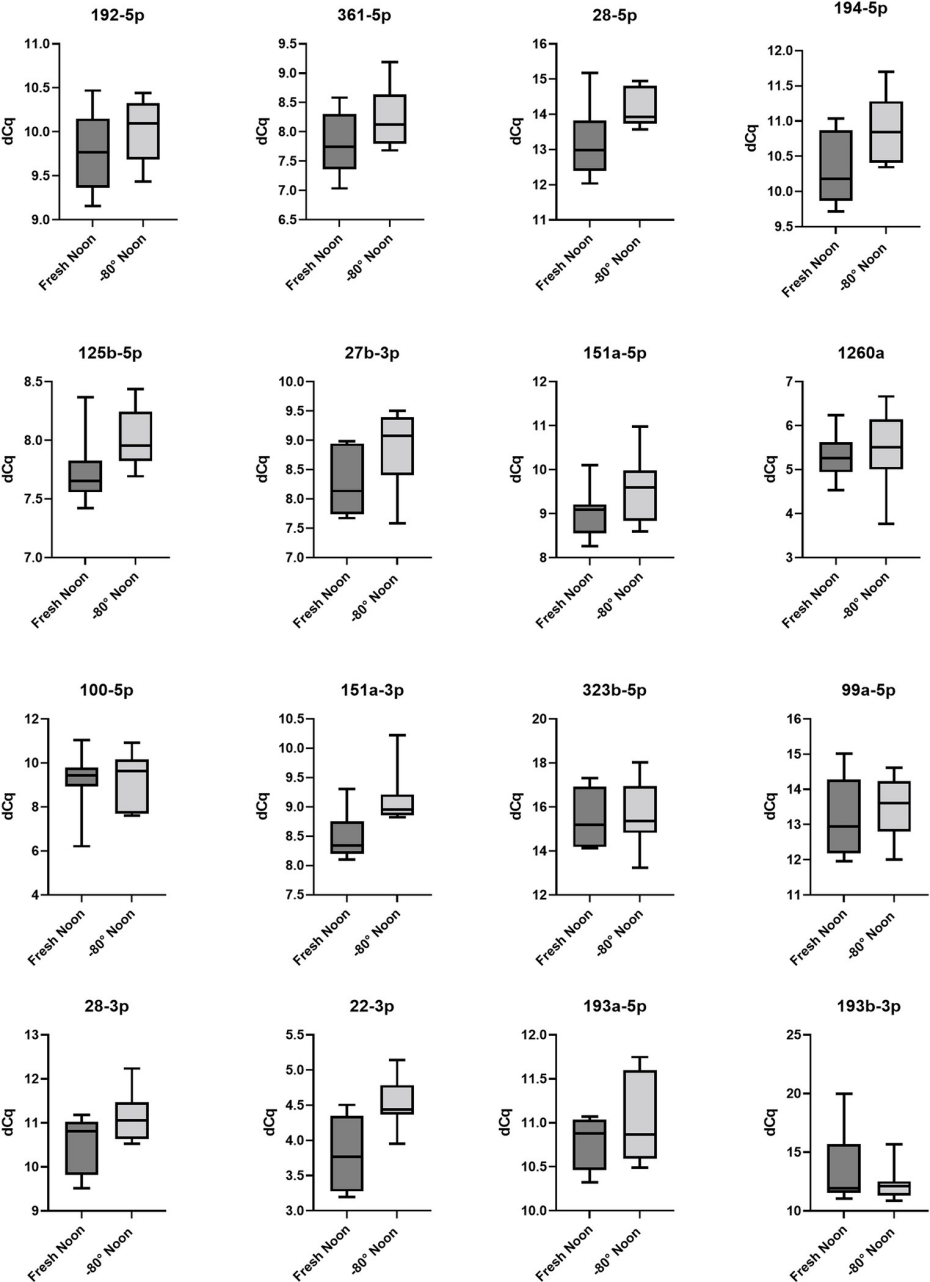
Fresh noon versus -80°C noon. Box plots of dCq values of serum MiRNA samples taken in the afternoon compares the analysis of samples (n = 8) within 24 hours and the analysis of samples (n = 8) after 14 days at -80°C. The expression of MiRNAs 28-5p, 194-5p, 151a-3p, 22-3p was lower after being stored at -80° for 14 days. The expression of miRNA 22-3p was significantly lower (p<0,01).

Figs 3 and 4 show the influence of differential storage conditions on the expression of the miRNA assays in serum. The complete panel of the selected 16 miRNAs samples stored for 14 days at -80° was detectable in serum. All 16 miRNAs investigated in this pilot study show a lower dCq expression after storage in general. Furthermore, all miRNAs show a lower detection value after being stored at -80°C for 14 days compared to the samples stored at 4°C and isolated within 24 hours.

We can demonstrate a slightly lower dCq expression value of 5 miRNAs in the morning samples, which were stored at -80°C for 14 days compared to the samples stored at 4°C and isolated within 24 hours. Nevertheless, these differences were not significant (p<0,01). Lower dCq expression was detected for miRNAs 194-5p (10.09 vs. 10.90; p = 0.0412), 27b-3p (8.112 vs. 8.944; p = 0.0241), 151a-5p (8.700 vs. 9.264; p = 0.0210), 100-5p (9.388 vs. 10.230; p = 0.0264) and 22-3p (3.660 vs. 4.411; p = 0.0182) (Fig 3).

Our data showed lower mean expression of 4 miRNAs from the panel of 16 miRNAs assays in the samples which were taken at noon und stored for 14 days at -80°C compared to the samples which were stored at 4°C and isolated within 24 hours. The lower mean expression has been shown for miRNAs 28-5p (13.18 vs. 14.16; p = 0.0312), 194-5p (10.29 vs. 10.88; p = 0.0349), 151a-3p (8.486 vs. 9.133; p = 0.0100) and 22-3p (3.821 vs. 4.530; p = 0.0069) (Fig 4). Nevertheless, only for miRNA 22-3p mean expression was significantly lowered (p <0,01). Except for miRNA 22-3p, the slightly lower expression dCq values after storage of samples at -80°C for 14 days was without statistical significance.

**Fig 5:**
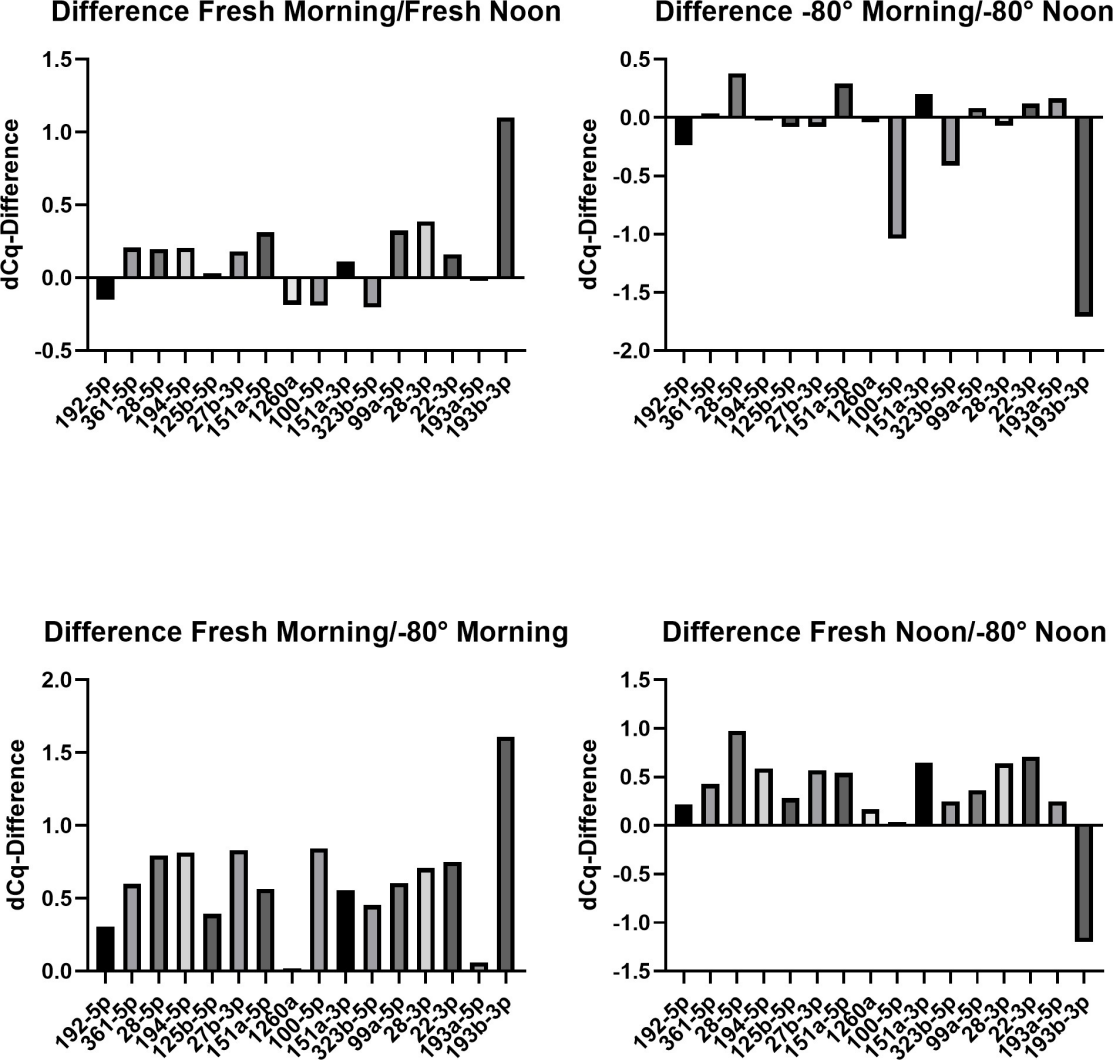
dCq differences. The dCq difference of all miRNA samples taken either in the morning under fasting condition and in the afternoon after normal eating and drinking. The dCq difference of the samples analyzed within 24h, as well as of the samples which were analyzed after 14 days storage at -80°C as shown. Overall, there are small differences under 1 dCq. Just the miRNA 193b-3p shows differences in all samples more than 1 dCq and the sample of the difference -80°C morning/ -80°C noon by miRNA 100-5p shows the difference more than 1dCq.

Overall, there were small dCq mean differences in the panel of selected 16 miRNAs (Fig 5). Only the miRNA 193b-3p shows differences more than 1 dCq in all samples and the miRNA 100-5p shows difference above 1 dCq in the sample in the difference -80°C morning/ -80°C noon. Except for miRNA 193b-3p, these differences have no statistical significance.

## Discussion

MiRNA and its use as a marker for cancer diagnosis and therapy monitoring as a minimally invasive liquid biopsy has a great potential in future oncology. This pilot study addresses the issues of sample collection in clinical practice—the different timepoints per day a given sample is collected and sample storage issues.

In this study, the miRNA profile in healthy individuals was shown to be overall very stable. We detected a slightly but not significantly lower expression of 16 miRNA assays after -80°C storage. Just one miRNA assay of 16 showed a significant difference (p<0,01). The miRNA expression, within the panel which was investigated in this study, did not differ between fasting and postprandial sampling.

Here, we found no significant difference when investigating the effect of fasting on serum miRNA expression. MacLellan et al. [18]. assessed the effect of fasting on serum miRNAs detectability in 7 healthy volunteers. The total miRNA count was slightly higher in the non-fasting samples compared to the fasting samples, although not significant. The findings in the study of MacLellan et al. [18] were detected in the samples, which were stored at -80°C. We can confirm these results and in addition also in the samples after short-term storage at 4°C with subsequent microRNA isolation. The fasting status alters the amount of lipids in the blood, which could interfere with RNA extraction and lead to variable miRNA levels [19]. The findings of MacLellan et al. [18] show that there is no significant difference in the miRNAs detected in fasting versus nonfasting samples demonstrating that the presence of lipids in the blood does not lead to the loss of less abundant miRNAs during extraction. In addition, no miRNAs examined in this publication were significantly differentially expressed between fasting and non-fasting samples, suggesting that fasting status will not interfere with the detection of serum miRNA biomarker in subjects with normal miRNA physiology.

Previous studies have shown that most circulating microRNAs are stable [20], but other authors [21] have published results of varying stability of microRNA and their degradation at room temperature. The reason given is the different resistance of different miRNAs in serum to RNase A during longer incubation. Köberle et al. [21] confirmed this hypothesis by adding RNase inhibitor to the tubes before blood collection. RNase inhibitor almost completely prevented the loss of investigated miRNAs. RNase inhibitor could be very useful for preserving cell-free circulating miRNAs and may allow their analysis and the discovery of novel miRNAs as biomarkers, which are usually escaping detection due to their rapid degradation.

Yamada et al. [22] reported that the concentration of circulating microRNA in samples stored at 4°C decreased significantly after 7 days. In our study, samples that were stored at 4°C and analysed within 24 hours are therefore not expected to have significant miRNA degradation. Matias-Garcia et al. [23] investigated in their study the influence of long-term storage on levels of eight circulating miRNAs. Their results show that the detected levels of most studied miRNAs showed no significant changes due to storage at ultra-low temperatures for up to 17 years. Matias-Garcia et al. [23] demonstrated a freeze-thawing effect in 8 examined miRNAs by freezing-thawing in one to four cycles. Significantly lower levels were shown for only one miRNA (30c-5p) after three and four sequential freezing-thawing cycles. Freezing samples at -80°C for 14 days showed a little effect on the concentration of the miRNAs we examined. Only miRNA 22-3p shows significant lower expression (p<0,01) after storage of samples for 14 days at -80°C. This miRNA is probably not suitable as a marker for samples stored in a biobank at -80°C. These results support the possibility of using samples from biobanks and allow the collection of samples over a longer period of time which is a huge advantage during clinical everyday practice.

A limitation of the present pilot study is the limited number of patients. The samples were stored at -80°C for only two weeks. In clinical practice, the storage time before use is usually longer. The results of miRNA stability in serum for long-term stored samples cannot be presented in this study and need to be investigated.

The strengths of this pilot study are that the selected miRNAs have been shown to be stable for clinical use, particularly as a control group in healthy individuals. The method of diagnosis of these concentrations is consistent with the conditions under which the samples are routinely handled.

In conclusion, our study shows that miRNAs have great potential in clinical applications. It is potentially an additional stable tool in laboratory diagnostics. MiRNAs proved to be a stable parameter in a cohort of patients. Concentrations did not differ over the time of day. We could observe just a marginal degradation of samples after storage at -80°C. The stability of miRNAs in healthy individuals shows their potential as a control group in the search for markers in oncology and in the diagnosis of other diseases.

## Supporting information

S1 Table Minimal data set. (XLSX)
